# Acetyltransferase P300 Regulates Glucose Metabolic Reprogramming through Catalyzing Succinylation in Lung Cancer

**DOI:** 10.3390/ijms25021057

**Published:** 2024-01-15

**Authors:** Qingzhi Ma, Qingmei Zeng, Kun Wang, Meirui Qian, Jingzhuo Li, Hao Wang, Huijie Zhang, Jianli Jiang, Zhinan Chen, Wan Huang

**Affiliations:** Department of Cell Biology, National Translational Science Center for Molecular Medicine, Fourth Military Medical University, Xi’an 710032, China

**Keywords:** *EP300*, lysine succinylation, *PGK1*, glucose metabolism, lung cancer

## Abstract

Aberrant protein post-translational modification is a hallmark of malignant tumors. Lysine succinylation (Ksucc) plays a vital role in cell energy metabolism in various cancers. However, whether succinylation can be catalyzed by acetyltransferase p300 remains unclear. In this study, we unveiled that p300 is a “writer” for succinylation, and p300-mediated Ksucc promotes cell glycometabolism in lung adenocarcinoma (LUAD). Specifically, our succinylome data revealed that *EP300* deficiency leads to the systemic reduction of Ksucc, and 79.55% of the p300-succinylated proteins were found in the cytoplasm, which were primarily enriched in the carbohydrate metabolism process. Interestingly, deleting *EP300* led to a notable decrease in Ksucc levels on several glycolytic enzymes, especially Phosphoglycerate Kinase 1 (*PGK1*). Mutation of the succinylated site of *PGK1* notably hindered cell glycolysis and lactic acid excretion. Metabolomics in vivo indicated that p300-caused metabolic reprogramming was mainly attributed to the altered carbohydrate metabolism. In addition, 89.35% of LUAD patients exhibited cytoplasmic localization of p300, with higher levels in tumor tissues than adjacent normal tissues. High levels of p300 correlated with advanced tumor stages and poor prognosis of LUAD patients. Briefly, we disclose the activity of p300 to catalyze succinylation, which contributes to cell glucose metabolic reprogramming and malignant progression of lung cancer.

## 1. Introduction

Lung cancer remains the leading cause of global cancer mortalities due to its propensity for invasion and metastasis [1]. Non-small cell lung cancer (NSCLC) accounts for 80% to 85% of all lung cancer cases, with lung adenocarcinoma (LUAD) being the most common histological subtype [1,2]. LUAD is characterized by a continuously high incidence of early metastasis, making early prediction and diagnosis challenging [3]. Therefore, there is an urgent need to develop systemic therapies targeting novel tumor markers and drug targets for the early detection and precise treatment of LUAD.

Glycometabolic reprogramming is recognized as a critical mechanism contributing to tumorigenesis and tumor progression [4]. Glycolysis dominates the energy metabolism of the rapidly proliferating tumor cells and is regulated by diverse factors [5]. Protein post-translational modifications (PTMs), such as phosphorylation [6,7] and lysine acetylation (Kac), are known to swiftly and widely modulate the glycometabolism of malignant cells [8].

Lysine succinylation (Ksucc) was recently identified as a novel PTM and another widespread acylation-like acetylation [9]. However, Ksucc differs from acetylation as it involves the conjugation of lysine residues with large succinyl groups, leading to remarkable changes in protein physicochemical properties and functions [10,11]. In addition, Ksucc can occur in both histones and non-histone proteins and plays an integral role in the initiation and progression of various diseases, including inflammation [12], Alzheimer’s disease [11], ischemia-reperfusion injury [13], and tumor [14]. The existing studies have shown that Ksucc may participate in glucose metabolism, lipid metabolism, TCA cycle, etc. [15,16].

Numerous studies reported that abnormal expression and activation of succinyltransferases are implicated in promoting tumor progression, which makes these enzymes promising new tumor markers and potential targets for anti-tumor therapy [17,18,19,20]. Certain inhibitors that target protein succinyltransferases or desuccinylases were shown to be effective in inhibiting various types of cancer, such as prostate cancer [21], colorectal cancer [22], leukemia [23], lymphoma [23], neuroblastoma [24], and other malignant tumors [23], and some medications (such as CCS1477 and FT-7051) have undergone clinical trials [25,26]. Consequently, the identification of the biological function and molecular mechanism of succinyltransferases and succinylation expands our understanding of novel tumor markers and the developing of drug targets based on protein succinylation.

Acyltransferase p300 plays a critical role in tumor growth, acting as either a transcriptional coactivator [27,28] or acyltransferase, thus exerting comprehensive effects on diverse tumors [29,30]. Previous studies demonstrated that p300 acetylates the lysine of histones, regulating lipid metabolism. More recently, studies revealed that p300 mediates 2-hydroxyisobutyrylation and modulates the processes of glycolysis and lactate excretion in cancer cells [31]. However, the precise molecular mechanisms and the biological consequences underlying p300-mediated Ksucc remain unclear.

In our study, we intend to elucidate whether p300 can catalyze protein succinylation and explore the effects of p300-mediated succinylation on cell metabolism and the progression of lung adenocarcinoma. These findings may expand the understanding of p300’s role in post-translational modification and lay the foundation for promising anti-tumor drugs targeting Ksucc in LUAD.

## 2. Results

### 2.1. p300 Catalyzes Lysine Succinylation in LUAD Cells

**Figure 1:**
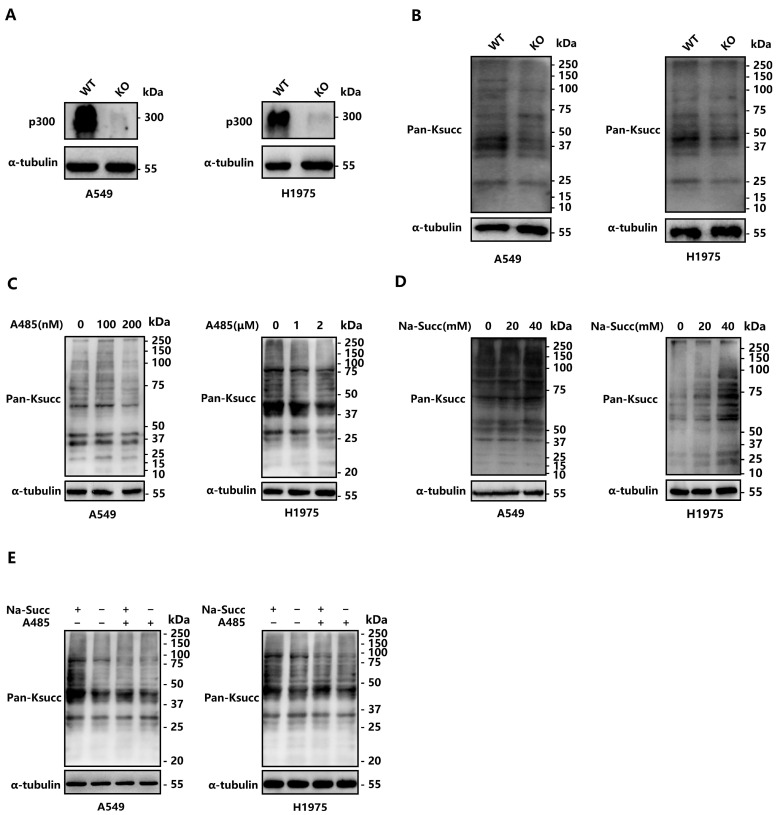
p300 catalyzes lysine succinylation in LUAD cells. (**A**,**B**) *EP300* deficiency impairs Kac and Ksucc in both A549 and H1975 cells. Kac and Ksucc levels in WT and *EP300* KO cells were determined by Western blot with a pan-antibody, respectively. (**C**) Ksucc levels of A549 and H1975 cells were inhibited by A485 in a dose-dependent manner. A549 and H1975 cells were incubated with A485 in increasing concentration. Ksucc levels were determined by Western blot with a pan-anti-succinyllysine antibody. (**D**) Sodium succinate enhanced Ksucc levels in A549 and H1975 cells in a dose-dependent manner. The levels of Ksucc were assessed by using a pan-anti-succinyllysine antibody in a Western blot after a culture procedure with sodium succinate. (**E**) p300-catalyzed succinylation was decreased by A485 and was enhanced by sodium succinate. WT and *EP300* KO cells (A549 and H1975) were treated with or without A485 and sodium succinate. Lysates of the cells above were analyzed by Western blot with a pan-anti-succinyllysine antibody.

To determine the role of *EP300* in succinylation, *EP300* KO lung adenocarcinoma cells (A549 and H1975) were constructed by using CRISPR/Cas9. The success of cell construction was confirmed by DNA sequencing, real-time PCR, and Western blotting analysis (Figure S1A–C). Then, both *EP300* WT cells and *EP300* KO cells were subjected to Western blot analysis using a pan-anti-acetylated-lysine antibody. The protein lysine acetylation (Kac) levels in LUAD cells were reduced upon the deletion of *EP300* (Figure 1A), correlating with p300’s catalytic activity of acetylation and providing a positive control. Consistently, Ksucc levels of LUAD cells, detected by a pan-anti-succinyllysine antibody, displayed a similar pattern in response to alterations in cellular p300 levels (Figure 1B). As shown in Figure 1B, the inhibitory impact of *EP300* knockout on succinylation was observed on both histones and non-histones.

To verify our findings above, we treated the human cell lines with a potent and selective catalytic inhibitor of p300: A485 [32]. Consistent with previous reports on its inhibitory effect on acetylation, A485 also inhibited p300 and decreased the Ksucc levels in both LUAD cells in a dose-dependent manner (Figure 1C). Additional immuno-blotting indicated that sodium succinate (Na-succ) promoted the p300-mediated Ksucc on various proteins (histones and non-histone proteins) in a dose-dependent manner (Figure 1D). However, the treatment with Na-succ, upon the addition of A485 to inhibit p300, failed to increase the succinylation, providing further evidence for the critical role of p300 in mediating Ksucc on different proteins (Figure 1E). In brief, p300 catalyzes the reaction of succinylation supported by Na-succ, and the inhibition of p300 strongly suppresses Ksucc in human LUAD cells.

### 2.2. Deletion of EP300 Decreases Ksucc Levels on Various Proteins

**Figure 2:**
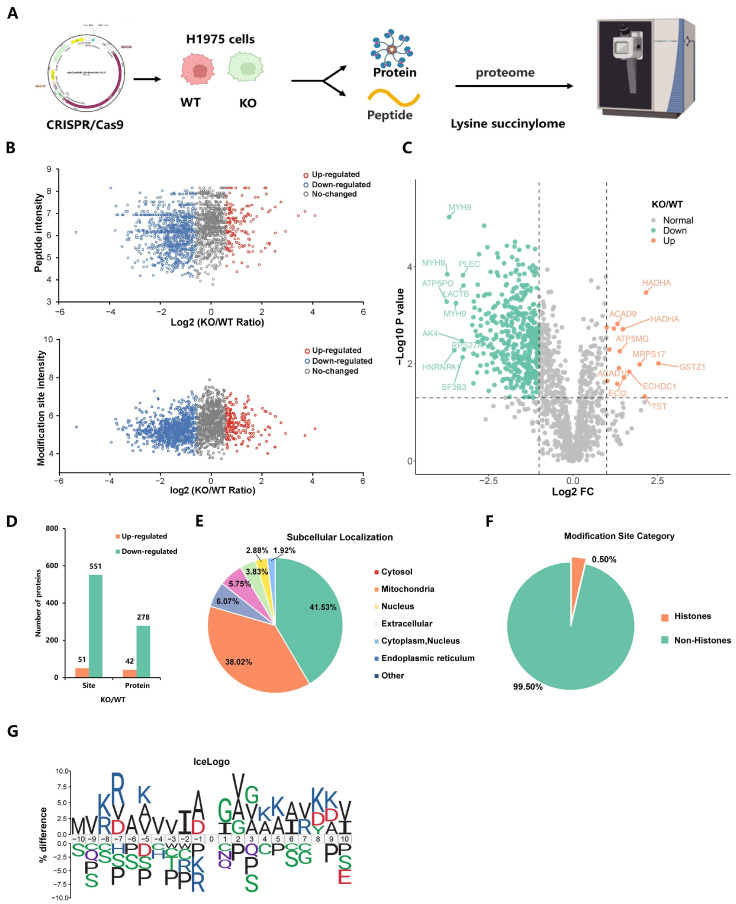
Deletion of *EP300* decreases Ksucc levels on various proteins. (**A**) Schematic representation of the experimental workflow for the quantitative succinylproteomics analysis. (**B**) The intensities of Ksucc sites and succinylated peptides with or without the loss of *EP300* are compared and shown in the scatter diagram. (**C**) Volcano plot showed that up-regulated and down-regulated Ksucc sites of LUAD cells in response to the deletion of *EP300* (*p*-value < 0.05). (**D**) The bar chart displayed the sites and proteins with differences in the difference analysis. (**E**) p300-mediated succinylation mainly occurred in the cytosol (41.53%) and mitochondria (38.02%) of LUAD cells. WolF Psort software (https://www.psort.org/, NAKAI Lab, Tokyo, Japan) was used to annotate the subcellular localization of proteins. (**F**) The pie chart indicated that more than 99% of Ksucc’s substrates were not histones. (**G**) The frequency of amino acids occurring near the Ksucc site is shown by the iceLogo. The size or color intensity of an amino acid reflects the difference in the frequency of the amino acid.

To better understand the landscape of the p300-catalyzed Kaucc, we performed a global quantitative succinylproteomics analysis with wild-type and *EP300*-knockout LUAD cells (Figure 2A and Figure S2A). All the samples for the quantitative succinylproteomics were confirmed by Coomassie Brilliant Blue Staining (Figure S2B) and validated by assessment procedures for repeatability (including Pearson’s Correlation Coefficient (PCC), Principal Component Analysis (PCA), and Relative Standard Deviation (RSD)) (Figure S2C–F). Then, we normalized the detected abundances of succinylated sites to the expression of their corresponding protein substrates and analyzed the intensities of Ksucc sites and succinylated peptides, revealing that *EP300* deficiency leads to the systemic reduction of Ksucc in LUAD cells (Figure 2B). Differential analysis showed a significant reduction in 278 proteins and 551 sites, while merely 42 proteins and 51 sites increased in response to the loss of *EP300* (Figure 2C,D and Figure S2G). An additional file shows the Ksucc sites in more detail (Additional File 1). Most of the identified p300-mediated Ksucc sites occurred in the cytosol (41.53%) and mitochondria (38.02%), suggesting a potential role of p300 in LUAD cell metabolism. (Figure 2E). Interestingly, 99.5% of the succinylated substrates were non-histones, implying that p300-catalyzed succinylation may not affect the transcription of genes in the nucleus (Figure 2F). Additionally, the flanking region close to Ksucc sites was examined for conserved sequence patterns to show p300’s biochemical preference and extrapolate Ksucc’s biological function. The iceLogo showed a preference for Ala at the −1 position, Gly at the +1 and +3 positions, and Val at the −4, −3, and +2 positions (Figure 2G). Arg is largely enriched at the −7 position but depleted at the −1 position. Overall, the p300-mediated catalytic reaction harnesses Lys but excludes Pro and Ser upstream and downstream near the Ksucc site. Motif analysis identified VK, AK, and KV motifs as significantly overrepresented for Ksucc sites (Motif Score > 9) (Figure S2H and Table S1).

### 2.3. p300-Mediated Ksucc Modulates Tumor Carbohydrate Metabolism

**Figure 3:**
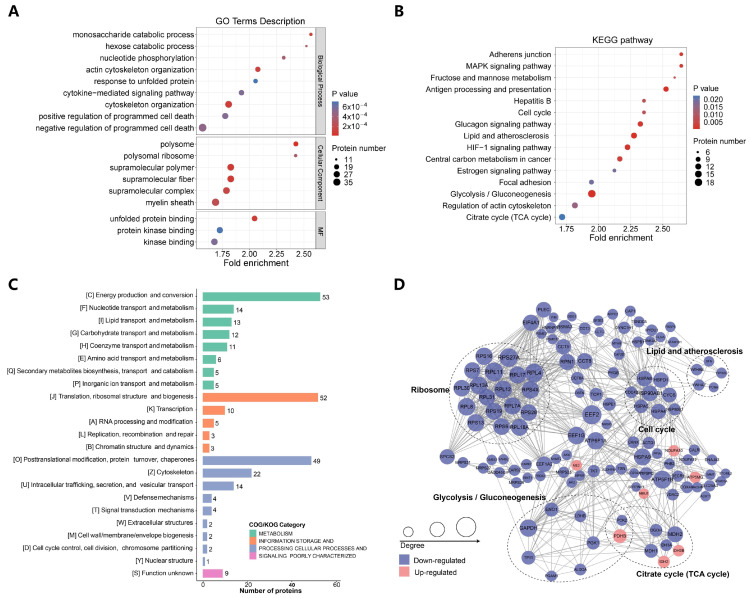
p300-mediated Ksucc modulates tumor carbohydrate metabolism. (**A**) p300-dependent Ksucc sites enriched in the metabolic process and protein binding. GO enrichment was conducted on the differential succinylation sites identified in Figure 2B,C. (**B**) p300-related Ksucc mainly targets glycolysis/gluconeogenesis in LUAD cells. KEGG pathway analysis proceeded based on the differential analysis of Ksucc sites. (**C**) The majority of succinylated proteins were classified into groups related to energy metabolism. Function categorization was performed by the KOGs database. (**D**) p300-mediated Ksucc specifically targets glycolytic enzymes. The interaction network is from the STRING database, and the size of the node positively corresponds to the degrees provided by STRING.

To further verify the function of succinylation catalyzed by p300, we carried out the GO enrichment analysis and discovered that p300-regulated Ksucc primarily enriched in metabolic process in terms of biological processes, supramolecular complex in terms of cellular component, and protein binding in terms of molecular function (MF) (Figure 3A). KEGG pathway enrichment analysis unveiled more precisely: the succinylation catalyzed by p300 mainly targets multiple metabolism-related pathways, such as fructose and mannose metabolism, central carbon metabolism in cancer, citrate cycle (TCA cycle), as well as glycolysis/gluconeogenesis (Figure 3B). We also searched the categories of Eukaryotic Orthologous Genes (KOGs) and found that most of the succinylated proteins were classified in the metabolism category, such as energy production and conversion (KOG category C) and carbohydrate metabolism and transport (KOG category G) (Figure 3C). Consistently, diverse functional groups, including glycolysis/gluconeogenesis, were displayed in the protein interaction network of succinylated proteomes built by the STRING database (Figure 3D).

### 2.4. p300 Succinylates Key Glycolytic Enzymes

**Figure 4:**
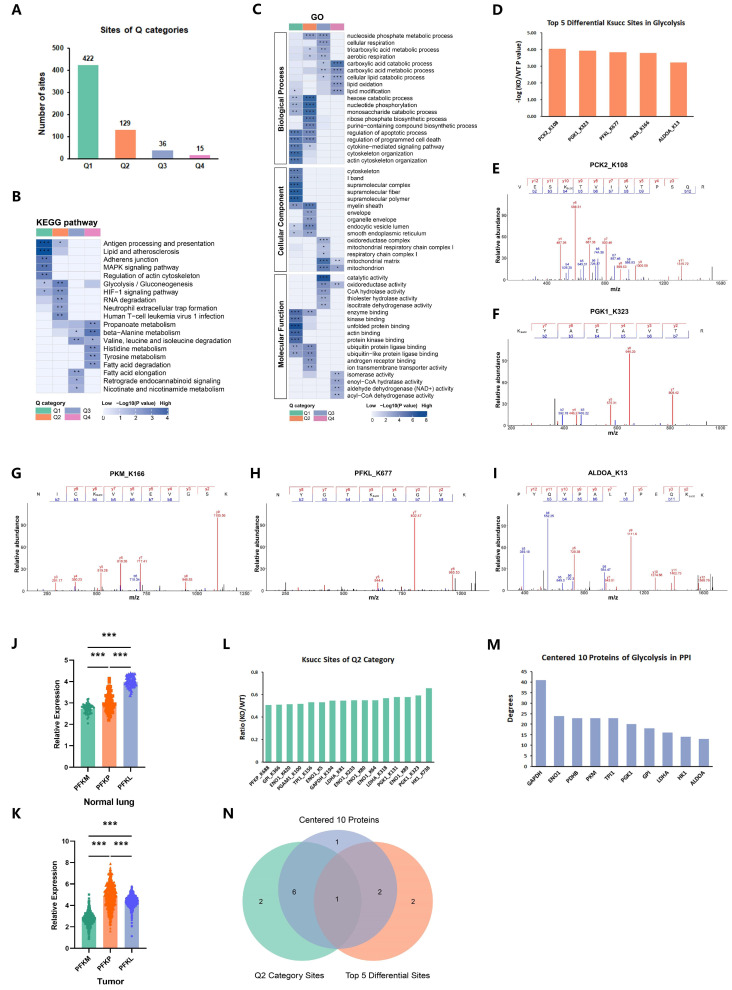
p300 succinylates key glycolytic enzymes. (**A**) The succinylated proteomes were subjected to a hierarchical clustering analysis and separated into four portions by *p*-value in the differential analysis, designated Q1 to Q4. (**B**,**C**) For each Q category, KEGG pathway (**B**) and GO categorization (**C**) enrichment were carried out (* *p* < 0.05, ** *p* < 0.01, *** *p* < 0.001). (**D**) Top five succinylated glycolytic enzymes catalyzed by p300. Shown are the top five Ksucc sites on glycolytic enzymes selected by *p*-value in differential analysis. (**E**–**I**) Mass spectrometry evidence of five succinylated sites (PCK2-K108succ, *PGK1*-K323succ, PFKL-K677succ, *PKM*-K166succ, and *ALDOA*-K13succ). (**J**,**K**) The bars showed the expression levels of the three subunits of PFK (PFKM, PFKP, PFKL) in normal lung tissues (*n* = 58, *** *p* < 0.001, values are expressed as mean ± SEM) and tumors. (**L**) Every succinyl modification site in the Q2 category in Figure 4A. (**M**) The top 10 proteins of glycolysis in the center of the PPI interaction network in Figure 3D. (**N**) The figure shows the overlap of succinylated proteins of the three ranges mentioned in Figure 4D,L,M (The green circle refers to the Ksucc sites in Figure 4L; The orange circle refers to the 5 Ksucc sites in Figure 4D; The blue circle refers to the 10 proteins in Figure 4M; The numbers in the colored areas represent the number of overlapping proteins).

To pinpoint the primary Ksucc-targeted proteins that drive glycolysis, we performed the hierarchical clustering analysis based on the differential analysis and divided the succinylated proteomes into four sections labeled Q1 to Q4 (Figure 4A). Then, GO classification and KEGG pathway enrichment were conducted for each Q group, respectively, which revealed that the succinylated proteins in the Q2 category participate in the glycolysis/gluconeogenesis (KEGG) and metabolic process (GO) (Figure 4B,C).

On the other hand, given that previous results that p300-catalyzed Ksucc primarily targeted glycolysis, we filtered the Ksucc sites involved in the pathway of glycolysis (KEGG) in the differential analysis and identified the top five Ksucc sites, which were Pyruvate Kinase M1/2 (*PKM*-K166), Phosphofructokinase, liver type (*PFKL*-K677), Phosphoenolpyruvate Carboxykinase 2, mitochondrial (PCK2-K108), Fructose-Bisphosphate Aldolase A (*ALDOA*-K13), and Phosphoglycerate Kinase 1 (*PGK1*-K323) (Figure 4D–I). We noticed that PCK2 is the only succinylated enzyme mainly involved in gluconeogenesis rather than glycolysis. Phosphofructokinase (PFK) is the first key enzyme in glycolysis and a complex tetrameric enzyme that contains three subtypes: platelet type (PFKP), muscle type (PFKM), and liver type (PFKL) [33,34]. To identify the main PFK in LUAD cells among the three subtypes, we downloaded RNAseq data of 510 LUAD patients from The Cancer Genome Atlas Program (TCGA) and compared the expression levels of the three subunits. In normal lung tissue, the expression of PFKL was the highest among the three subtypes. However, different from normal lung tissue, RNA sequencing data analysis showed that PFKP expression was the highest in LUAD (Figure 4J,K).

To further identify the most important succinyl modification site targeting glycolysis, we intersected the top 10 proteins of glycolysis in the center of the PPI interaction network in Figure 3D, all the succinyl modification sites in the Q2 category in Figure 4A, and the top five differential modification sites of glycolysis in Figure 4D. Then, we were surprised to find that *PGK1* presented simultaneously in the three previously described terms, which recommended that the occurrence of succinylation on *PGK1* lysine 323 may be a vital node in the mechanism of p300-dependent succinylation enhancing glycolysis (Figure 4M,N).

### 2.5. p300-Mediated PGK1-K323succ Enhances Tumor Glycolysis

**Figure 5:**
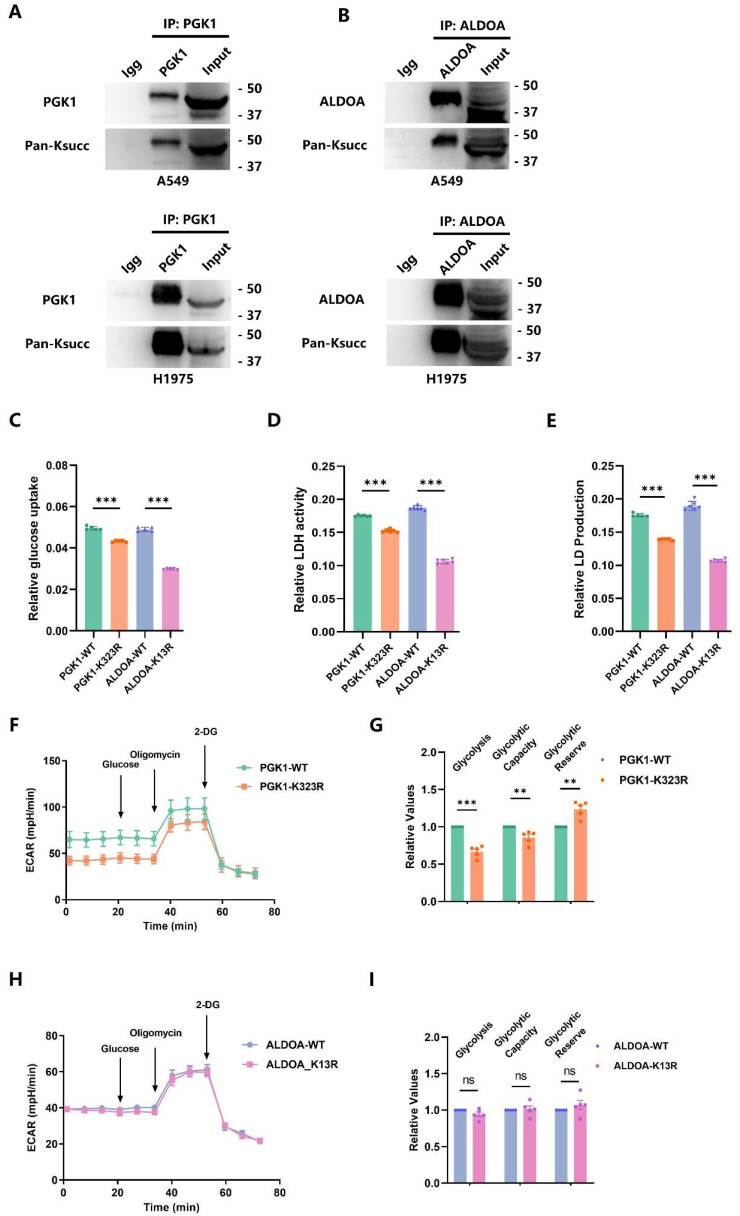
p300-mediated *PGK1*-K323succ enhances tumor glycolysis. (**A**,**B**) The Ksucc of *PGK1* and *ALDOA* were detected by IP assays and immunoblotting in cells (A549 or H1975) with indicated antibodies. (**C**–**E**) The glucose uptake, LDH activity, and lactic acid production of *PGK1*-WT and *ALDOA*-WT or *PGK1*-K323R and *ALDOA*-K13R LUAD cells were detected by indicated kits (*n* = 5–6, *** *p* < 0.001, values are expressed as mean ± SEM). (**F**) The seahorse analysis showed that *PGK1*-K323R reduces the extracellular acidification of LUAD cells compared to *PGK1*-WT. (**G**) The bar plot showed the relative glycolysis, glycolytic capacity, and glycolytic reserve of *PGK1*-WT and *PGK1*-K323R in seahorse results (*n* = 5, ** *p* < 0.01, *** *p* < 0.001, values are expressed as mean ± SEM). (**H**) The seahorse analysis showed that *ALDOA*-K13R cannot reduce the extracellular acidification of LUAD cells compared to *ALDOA*-WT. (**I**) The bar plot showed the relative glycolysis, glycolytic capacity, and glycolytic reserve of *ALDOA*-WT and *ALDOA*-K13R in seahorse results (*n* = 5, ns, no significance, values are expressed as mean ± SEM).

We also performed an Immunoprecipitation (IP) test using corresponding monoclonal antibodies to obtain the purified enzyme samples from cell lysates of A549 and H1975, which were then subjected to Western blot experiments to verify the presence of the Ksucc of each enzyme. Coinciding with the data of mass spectrometry, succinylation was observed on *PGK1* and *ALDOA* in both A549 and H1975 cells (Figure 5A,B). Furthermore, we created the LUAD cells with stable point mutations (*PGK1*-K323R, *ALDOA*-K13R) and replicated the functional experiments on glycolysis in vitro to verify the Ksucc-corresponding functions. Subsequently, we found that both *PGK1*-K323R and *ALDOA*-K13R decreased glucose uptake, LDH activity, and lactate production in LUAD cells (Figure 5C–E). On the other hand, the mutation of *PGK1* K323 attenuated the extracellular acidification in the seahorse test, reduced the glycolysis and glycolytic capacity, but increased the glycolytic reserve of LUAD cells (Figure 5F,G). Unlike *PGK1*, the *ALDOA* K13 mutant failed to reduce the extracellular acidification, glycolysis, glycolytic capacity, and glycolytic reserve in malignant cells (Figure 5H,I). So, it was found that p300-strengthened glycolysis and lactic acid production in LUAD cells is, to a large extent, driven by the *PGK1*-K323succ.

### 2.6. EP300 Knockout Inhibits Cell Energy Metabolism, Particularly Carbohydrate Metabolism In Vivo

**Figure 6:**
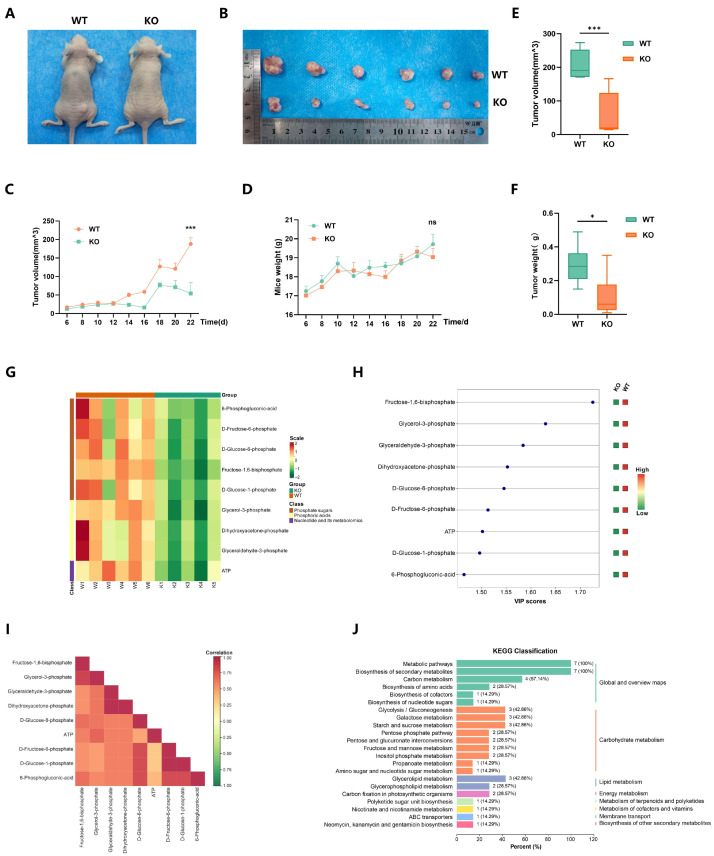
*EP300* knockout inhibits cell energy metabolism, particularly carbohydrate metabolism in vivo. (**A**,**B**) A tumor formation experiment in mice was performed by injecting cells (A549) with or without *EP300* knockout into nude mice (Group WT, *n* = 6 mice; Group KO, *n* = 6 mice). (**C**,**D**) Quantification of the volume (**G**) and weight (**H**) of tumors formed in Figure 6E,F. (ns, no significance, *** *p* < 0.001, values are expressed as mean ± SEM; Group WT, *n* = 6 mice; Group KO, *n* = 6 mice). (**E**,**F**) Quantification of the volume (**I**) and weight (**J**) of isolated tumors formed in (**E**,**F**). (* *p* < 0.05, *** *p* < 0.001; Group WT, *n* = 6 mice; Group KO, *n* = 6 mice). (**G**) Clustering heatmap of differential metabolites. Horizontal is the sample name, vertical is the metabolite information, and scale is the expression amount obtained after standardized treatment (the red color, the higher the expression amount; the green color, the lower the expression amount) (Group WT, *n* = 6 mice; Group KO, *n* = 5 mice, one case (K6) in the Group KO was excluded by PCA and OPLS-DA). (**H**) VIP value of differential metabolites. The horizontal coordinate is the VIP value, the vertical coordinate is the metabolite, and the green represents the down-regulated differential metabolite. (**I**) Horizontal is the name of differential metabolite; longitudinally, it is the name of differential metabolite. Red indicates a strong positive correlation, and green indicates a strong negative correlation; the darker the color, the larger the absolute value of the correlation coefficient between samples. (**J**) KEGG classification of differential metabolites. The ordinate is the term of the KEGG pathway, the number in the figure indicates the number of differential metabolites annotated to this pathway, and the ratio of the number of differential metabolites annotated to this pathway to all the differential metabolites annotated to KEGG is in parentheses.

To obtain in vivo proof, we injected WT and *EP300*-KO cells subcutaneously in nude mice to create tumor-bearing mice models (Figure 6A,B). Then, tumor volume and weight data were collected on live mice (Figure 6C,D) and isolated tumors (Figure 6E,F), respectively. As expected, the results of tumor formation experiments suggested that *EP300* knockout surely suppressed the tumor growth of LUAD.

With tumor tissues obtained from the models above, we performed metabolomics targeting energy metabolism using mice LUAD models from *EP300*-KO and WT tumor tissues. Orthometric Partial Least Squares-Discriminant Analysis (OPLS-DA) and Principal Component Analysis (PCA) of samples (including quality control samples) were carried out to explore the overall metabolic differences between samples of each group and the variability between samples within the group (Figure S3A,B). The metabolomics project quantified forty-five metabolites and identified nine differential metabolites compared between the KO and WT groups (Figure 6G and Figure S3C). An additional file shows the metabolites in more detail (Additional File 2). All the differential metabolites are present in the process of glycolysis, and their specific concentrations in different groups are shown in the violin diagram (Figure S3D). The variable importance in the projection (VIP) value of fructose-1,6-bisphosphate was the highest among the nine differential metabolites (Figure 6H). KEGG enrichment also verified that all the differential metabolites (100%) were distributed in metabolic pathways, 57.14% in carbon metabolism, and 42.86% in glycolysis/gluconeogenesis (Figure 6I). The Pearson Correlation Plot and Chord Diagram represented the metabolic proximities between metabolites with significant differences (Figure 6J and Figure S3E).

### 2.7. EP300 Deficiency Impairs Glycolysis and Malignant Phenotypes of LUAD Cells

**Figure 7:**
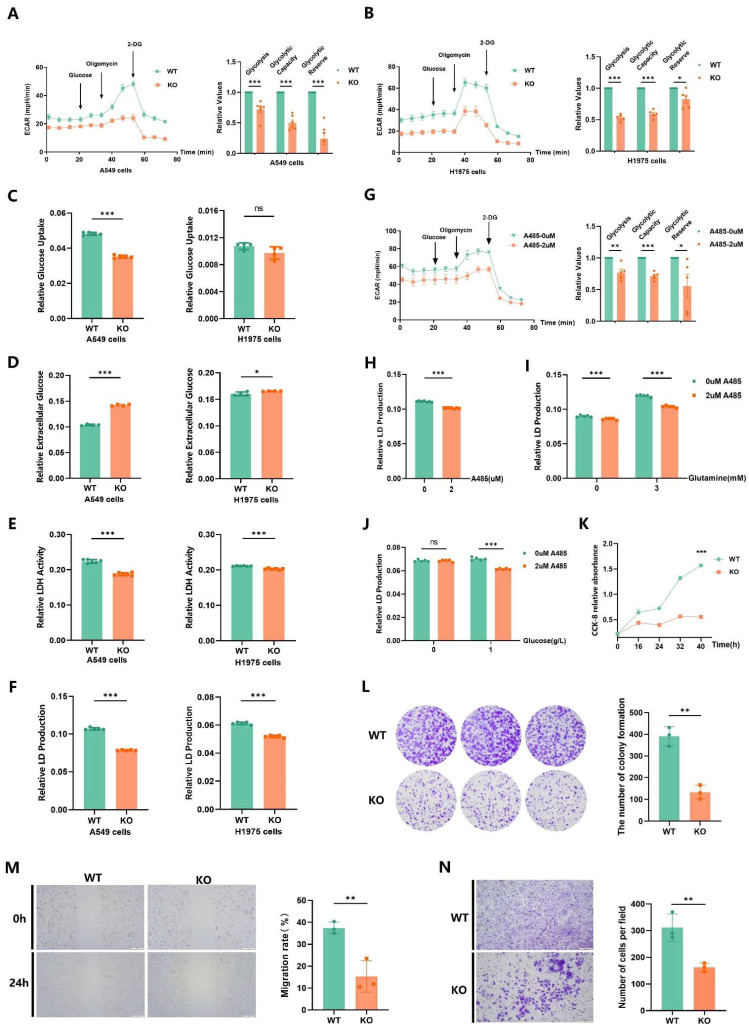
*EP300* deficiency impairs glycolysis and malignant phenotypes of LUAD cells. (**A**,**B**) *EP300* deficiency of A549 and H1975 cells reduces extracellular acidification. The bar charts represent glycolysis, glycolytic capacity, and glycolytic reserve, respectively (*n* = 4–6, ns, no significance, * *p* < 0.05, *** *p* < 0.001, values are expressed as mean ± SEM). (**C**,**D**) *EP300* deficiency of A549 and H1975 cells reduces the glucose uptake and increases the extracellular glucose concentrations (*n* = 4–5, * *p* < 0.05, *** *p* < 0.001, values are expressed as mean ± SEM). (**E**,**F**) *EP300* deficiency of A549 and H1975 cells reduces the LDH activity and lactic acid production (*n* = 5–6, *** *p* < 0.001, values are expressed as mean ± SEM). (**G**) Adding the inhibitor of p300 reduces the extracellular acidification as well (*n* = 5, * *p* < 0.05, ** *p* < 0.01, *** *p* < 0.001, values are expressed as mean ± SEM). (**H**) Adding the inhibitor of p300 reduces the lactic acid production as expected (*n* = 7, *** *p* < 0.001, values are expressed as mean ± SEM). (**I**,**J**) Adding the inhibitor of p300 reduces the Lactic acid production with or without treatment of glutamine or glucose. Cells incubated in a medium without glucose and glutamine were treated with extra glutamine or glucose in the presence of A485 (*n* = 5, *** *p* < 0.001, values are expressed as mean ± SEM). (**K**–**N**) *EP300* deficiency suppresses the proliferation (**A**), stemness (**B**), migration (**C**), and invasion (**D**) of LUAD cells. (**K**). Cells were plated in a complete medium and switched to the indicated medium at 0 h (*n* = 5, *** *p* < 0.001, values are expressed as mean ± SEM). (**L**) Cells were plated in a complete medium and dyed with crystal violet. The magnification of the microscope is ×100. The number of formed colonies was provided by ImageJ 1.51 software. (*n* = 3, ** *p* < 0.01, values are expressed as mean ± SEM). (**M**) Cells were completely attached to the wall and plated in a complete medium. Migration rates were provided by ImageJ 1.51 software. (*n* = 3, ** *p* < 0.01, values are expressed as mean ± SEM). (**N**) After 48 h of incubation in the Transwell chamber, cells were fixed with glutaraldehyde and stained with crystal violet. Cells in the lower chamber were analyzed by ImageJ 1.51 software. (*n* = 3, ** *p* < 0.01, values are expressed as mean ± SEM).

By measuring the Extracellular Acidification Rate (ECAR), we assessed the strength of glycolysis in A549 and H1975 cells and proved that the loss of *EP300* can result in impaired glycolysis in LUAD (Figure 7A,B, left). As shown in the bar plot, *EP300* knockout led to damages in basal glycolysis, glycolytic capacity, and glycolytic reserve (Figure 7A,B, right). In line with the observations above, the other experiments testing glycolysis also showed that the *EP300* deficiency is linked to a reduction in glucose uptake, LDH activity, and lactic acid production in tumor cells but increased extracellular glucose in the tumor microenvironment (TME) (Figure 7C–F). Noteworthily, only the *EP300* defect of H1975 cells cannot generate an obvious alteration in glucose uptake, which may be attributed to the heterogeneity of *EP300*-KO cells and the variance of glucose uptake. (Figure 7C, right). Using the p300 inhibitor A485 as a variable, we examined the ECAR and lactic acid again and found that the impact of A485 on glycolysis resembles that of *EP300* knockout (Figure 7G,H). To identify the connection between substrates for glycolysis and p300, we quantified lactic acid production of LUAD cells in the presence or absence of appropriate concentrations of energy substrates. It turned out that A485 inhibited the glycolysis regardless of the availability of glutamine, but A485 inhibited the glycolysis only when glucose was present (Figure 7I,J). These observations raised the intriguing hypothesis that p300-altered glycolysis relies on glucose. So far, we have validated that the defect of *EP300* inhibits glucose-related glycolysis in LUAD cells independently.

To further evaluate the contribution of *EP300* to metabolic regulation of malignancies of LUAD cells, we performed standard experimental procedures, including cell count assay, colony formation assay, scratch test, and transwell invasion assay in vitro to assess the effects of the loss of *EP300*. The results indicated that *EP300* deletion has a considerable negative effect on tumor proliferation, stemness, migration, and invasion, thus indicating that *EP300*-enhanced glycolysis is essential for the maintenance of tumor malignant phenotype (Figure 7K–N).

### 2.8. EP300 Coincides with Advanced Tumor Stage and Poor Prognosis of LUAD Individuals

**Figure 8:**
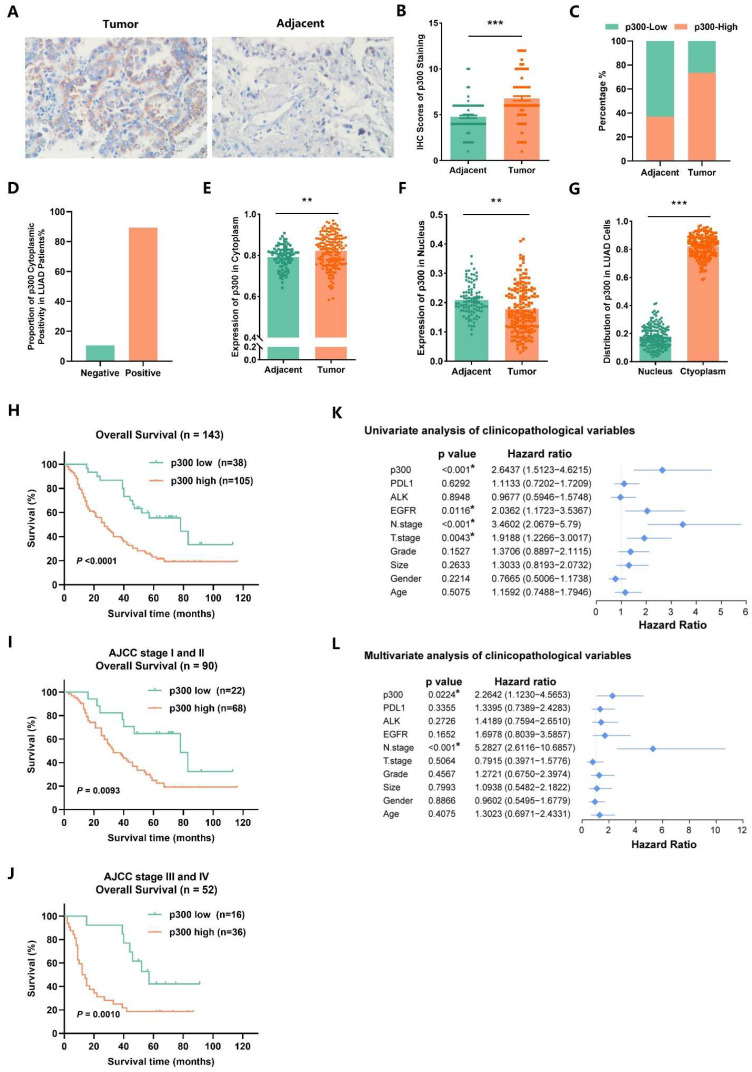
*EP300* coincides with advanced tumor stages and poor prognosis of LUAD individuals. (**A**) The expression of *EP300* in tumors was higher than that in adjacent tissues. The expression of *EP300* in tumors and adjacent tissues was determined by IHC staining; scale bar, 29.34 μm. (**B**) The scatter bar plot shows the IHC scores of overall p300 expression, and the paired *t*-test was used (n of pairs = 125, *** *p* < 0.001). (**C**) The stacked bar plot displays the proportion of p300-positive individuals in tumors and adjacent tissues. (**D**) LUAD patients with p300 cytoplasmic localization positivity accounted for 89.35% (*n* = 169). (**E**,**F**) The bar chart with scatter points suggests that p300 is mainly located in the cytoplasm in tumors but in the nucleus in adjacent lung tissues (Tumor, *n* = 169; Adjacent, *n* = 93, ** *p* < 0.01). (**G**) In LUAD cells, p300 is mainly located in the cytoplasm (*n* = 169, *** *p* < 0.001). (**H**) OS (overall survival) rates of all individuals with high or low *EP300* expression levels were determined by Kaplan–Meier analysis (*n* = 143). (**I**) Kaplan–Meier analysis of patients with early-stage LUAD. AJCC stages I and II were identified as early stages (*n* = 90). (**J**) Kaplan–Meier analysis of patients with late-stage LUAD. AJCC stages III and IV were identified as late-stage, and one individual lost the staging information (*n* = 52). (**K**,**L**) Univariate and multivariate COX regression analyses of *EP300* and other clinicopathological variables (*n* = 143, * *p* < 0.05, values are expressed as mean ± SEM).

To investigate the expression of *EP300* in LUAD patients, we carried out IHC analysis in tissue microarrays of tumors and their matched adjacent normal tissues (Figure 8A). Compared to adjacent tissues, tumors displayed much higher IHC scores of overall p300 staining and a higher proportion of p300-positive cases (Figure 8B,C). To identify the roles of *EP300* in LUAD progression, we analyzed the correlation between the expression of *EP300* and various clinicopathological variables of patients with LUAD using chi-square tests. No significant correlation was found between the expression of *EP300* and gender, age, Programmed Cell Death 1 Ligand 1 (*PDL1*) expression, Anaplastic Lymphoma Receptor Tyrosine Kinase (*ALK*) expression, tumor size, tumor differentiation, and N stage. However, high expression of *EP300* was correlated with high *EGFR* expression (*p* = 0.037) and advanced T stage (*p* = 0.048) (Table 1). Next, by analyzing the subcellular localization of p300 in the tumor tissues from LUAD patients, a positive rate of 89.35% of p300 cytoplasmic expression was detected (Figure 8D). By quantifying p300 expression in cytoplasm and nucleus, respectively, and comparing to adjacent normal tissues, the cytoplasmic expression level of p300 in tumor tissues was considerably higher (*p* < 0.01) (Figure 8E). However, adjacent normal tissues had much higher nuclear p300 expression than tumor tissues (*p* < 0.01) (Figure 8F). Further comparison of the expression intensity of p300 in cytoplasm and nucleus showed that the expression of p300 in cytoplasm was significantly higher than that in the nucleus (*p* < 0.001) (Figure 8G). Moreover, Kaplan–Meier analysis showed that high *EP300* expression levels in LUAD patients were significantly correlated with poor overall survival (OS) (*p* < 0.0001) (Figure 8H). This correlation between *EP300* expression and prognosis was found in both early stages (AJCC stages I and II, *p* = 0.0093) and advanced stages (AJCC stages III and IV, *p* = 0.0010) of LUAD, exhibiting the prognostic significance of *EP300* for both early and advanced stage LUAD patients (Figure 8I,J). Then, we assessed the significance of *EP300* for the prognosis of LUAD by conducting Cox regression analysis of covariates, including gender, age, tumor size, tumor grade, T and N staging, and expression of *PDL1*, *ALK*, *EGFR*, and *EP300*. The results of the univariate analysis suggested that T staging, N staging, *EGFR* expression, and *EP300* expression are related to the prognosis of LUAD patients, and further multivariate analysis suggested that N staging (*p* < 0.001) and *EP300* expression (*p* = 0.0224) could be independent prognostic factors for individuals with LUAD (Figure 8K,L).

## 3. Discussion

Metabolic reprogramming is a hallmark of malignancy. One of the key alterations observed in cancer cells is their shift towards enhanced glycolysis to meet the increased bioenergetic and biosynthetic demand [35,36]. Accumulating evidence suggested that glycolytic disruptions and the resulting acidic tumor environment have a crucial role in the genesis and progression of lung cancer [37,38,39,40]. Ksucc is a kind of PTM closely related to cell metabolism [41,42,43]. Succinyl-CoA, the main substrate of succinylation, can be generated by the tricarboxylic acid cycle and lipid and amino acid metabolism. On the other hand, succinylation can drastically alter cell metabolism by regulating enzymes involved in metabolism [44]. Previous studies have demonstrated that *SIRT5* can mediate the desuccinylation of several mitochondrial proteins, such as glutamate dehydrogenase (*GDH*), malate dehydrogenase, and citrate synthase (*CS*), which, in turn, affect metabolic processes in tumor cells [45]. Despite these insights, no systematic succinylome analysis has been conducted to present the landscape of Ksucc in lung cancer, and the mechanism by which Ksucc regulates glucose metabolism in lung cancer remains unclear. In this study, we identify p300 as a key player in altering glucose metabolism directly by catalyzing the succinylation of glycolytic enzymes. Therefore, this novel finding sheds new light on the mechanism of glycometabolic reprogramming in lung cancer cells.

p300, an acyltransferase, possesses a broad range of catalytic activities for various PTMs, such as acetylation [46], propionylation [47], butyrylation [47], 2-hydroxy-isobutyrylation [31], crotonylation [48], and lactation [49]. Previous research has shown that lysine acetyltransferase 2A (*KAT2A*) and carnitine palmitoyltransferase 1A (*CPT1A*) are the main enzymes that catalyze succinylation [50]. Our study is the first to identify p300 as a writer of Ksucc. The earlier analysis of p300’s catalytic domain and flanking domains suggests that acyltransferase p300 mainly catalyzes the acylation of short-chain fatty acyl-CoA variants represented by acetylation and lactation [51,52]. In contrast, succinylation involves a long-chain, negatively charged, and acidic succinyl group. Despite these differences, we found that p300 is capable of catalyzing succinylation as well. Interestingly, our study revealed that 79.55% of succinylated peptides catalyzed by p300 were beyond the nucleus, and 99.5% of which were non-histone proteins. This is far different from the previously reported classic catalytic capacity of p300 in the nucleus. Acyltransferase p300 used to be considered as a predominantly nuclear protein, which can catalyze the acetylation of histones [53,54,55], recruit transcriptional complexes [56], and further modulate the transcriptional control of cancer-related genes. In LUAD, we demonstrated that the primary p300-mediated Ksucc occurs outside the nucleus, and the main effect of p300 is to enhance cellular energy metabolism, particularly glucose metabolism, thereby promoting the malignant phenotype of lung cancer cells. While previous reports had mainly identified succinylated substrates in mitochondria [57], our succinylome analysis revealed that most of the p300-related succinylated substrates were cytoplasmic proteins. Meanwhile, the pathological data of patients’ tissues showed that the majority of p300 is located in the cytoplasm rather than the nucleus. This suggests that p300-mediated succinylation is more likely to participate in metabolic processes located in the cytoplasm, such as glycolysis. A substantial amount of p300-catalyzed Ksucc was observed in mitochondria as well, which contrasts with the earlier claims that the mechanism of acylation modification in mitochondria was mainly spontaneous chemical reactions [58]. In conclusion, in LUAD, p300 is localized primarily in the cytoplasm and catalyzes Ksucc on cytoplasmic metabolic enzymes, which induces metabolic reprogramming more directly. This finding proves the significance of p300 in tumor post-translational modifications and provides a novel perspective on its catalytic function in cancer.

In our study, we identified p300-catalyzed succinylation on a key substrate, *PGK1*. *PGK1* catalyzes the only productive reaction in glycolysis other than *PKM*, transferring a phosphate group from 1,3-diphosphoglycerate to ADP, thus forming ATP and 3-phosphoglycerate. Our results of metabolomics suggested that p300-catalyzed Ksucc is closely related to the intracellular concentrations of glycerol-3-phosphate and glyceraldehyde 3-phosphate, which are metabolically close to 3-phosphoglycerate, the product of *PGK1*-mediated reaction in cell glycolysis. This finding is consistent with our previous functional experiments on *PGK1*-K323succ. *PGK1* also displays different types of PTMs under different conditions, such as phosphorylation [59], acetylation [60], and ubiquitination [61], which are closely related to *PGK1* activity and the malignant progression of tumors. The acetylation of *PGK1* K323 has been reported, with P300/CBP-associated factor (*PCAF*) and Sirtuin 7 (*SIRT7*) recognized as the enzymes regulating *PGK1* K323 acetylation in both directions [60]. In this study, we characterized p300 as the enzyme catalyzing *PGK1* K323 succinylation by succinylome analysis and Western blot assay. Functional experiments with the *PGK1* mutant (K323R) also indicated that *PGK1*-K323succ up-regulates the glycolysis of LUAD cells. Studies have shown that there is crosstalk between diverse modifications at the same amino acid residue of proteins. Furthermore, questions remain to be investigated: Are the acetylation and succinylation of *PGK1* K323 functionally competitive antagonists? Does p300 catalyze the different modifications of *PGK1* K323 in different pathologic and physiological states? How does Ksucc of *PGK1* K323 enhance glycolysis? Addressing these questions will be important for gaining a deeper understanding of the functional interplay between different PTMs at *PGK1* K323 and the role of p300 in regulating glycolysis.

Previous studies have implied that high *EP300* expression correlates with poor prognosis in hepatocellular carcinoma [62] and breast cancer [63]. However, the specific subcellular localization of p300 in lung cancer cells and whether it can also be used as a prognostic marker for lung cancer patients have not been investigated. In our study, immunohistochemical analysis of patients’ tumor tissues proved that p300 possesses different expression patterns in cancer and paracancer. Importantly, we found that p300 is predominantly localized in the cytoplasm rather than the nucleus in LUAD cells. Clinical analysis of this research discovered a correlation between *EP300* expression and advanced tumor stage in LUAD, suggesting *EP300* could be an independent prognostic factor for individuals with LUAD. Consequently, we identified p300, especially cytoplasmic, as a prognostic biomarker for predicting LUAD patient outcomes. Current drugs targeting *EP300* are too broad and untargeted, lacking specificity to suppress a particular cancer-related function of *EP300* [25,26,64,65]. Our study provides a new path for the development of distinctive molecular drugs specifically targeting cytoplasmic p300 and improvement of prognosis for survival outcomes in patients with lung cancer.

## 4. Materials and Methods

### 4.1. Key Resources Table

The reagents or resources involved in this study are listed in the table below (Table 2).

### 4.2. Cell Culture

The A549 and H1975 cells were obtained from the American Type Culture Collection (ATCC, Gaithersburg, MD, USA). All the cell lines were authenticated using Short Tandem Repeat DNA profiling and maintained in RPMI 1640 with 10% fetal bovine serum, 2 mM L-glutamine, 100 U/mL of penicillin, and 100 μg/mL of streptomycin at 37 °C and 5% CO_2_.

### 4.3. Cell Lines

*EP300* KO cells (A549 or H1975) were generated with a human *EP300* CRISPR/Cas9 KO plasmid from Sangon Biotech (Shanghai, China, target site (Sg3): CCGAGAATGTGGTGGAACCG). The deletion of *EP300* in single-cell clones was tested by DNA sequencing and Western blotting analysis.

A549 cells were used to generate cell lines stably overexpressing certain enzymes (A549-*PGK1*-WT, A549-*PKM*-WT, A549-*ALDOA*-WT) using recombinant plasmids as follows: pLV3-CMV-*PGK1*-3×FLAG-CopGFP, pLV3-CMV-*PKM*-3×FLAG-CopGFP, pLV3-CMV-*ALDOA*-3×FLAG-CopGFP. Cells with stable point mutations (A549-*PGK1*-K323R, A549-*PKM*-K166R, A549-*ALDOA*-K13R) were constructed from the overexpressing cells using recombinant plasmids as follows: pLV3-CMV-*PGK1*-K323R-3×FLAG-CopGFP, pLV3-CMV-*PKM*-K166R-3×FLAG-CopGFP, pLV3-CMV-*ALDOA*-K13R-3×FLAG-CopGFP. The overexpressing plasmids were provided by MiaoLingbio (Wuhan, China).

### 4.4. Western Blot and Antibodies

The cultured cells were washed with PBS and then treated with radioimmunoprecipitation assay lysis buffer (RIPA) containing phenylmethyl-sulfonyl fluoride (PMSF). Subsequently, cell samples were lysed for 10 min on ice, centrifuged at 16,000× *g* for 15 min, and the supernatant was collected. The BCA protein assay kit (23225, Thermo Fisher Scientific, Waltham, MA, USA) was used to quantify protein amounts. Equal amounts of protein were resolved on a 10% SDS-PAGE gel and then transferred to PVDF membranes (Millipore, Burlington, MA, USA). Membranes were incubated in 5% skim milk for 1 h at room temperature and then with primary antibodies diluted in blocking buffer at 4 °C overnight: Anti-p300 (Santa Cruz, sc-48343, dilution 1:1000), anti-tubulin (Proteintech, 66031-1-lg, dilution 1:5000), anti-succinyllysine (PTM Bio, PTM-419, dilution 1:1000), anti-*ALDOA* (Proteintech, 11217-1-AP), anti-*PGK1* (Proteintech, 17811-1-AP), anti-*PKM* (Proteintech, 25659-1-AP) antibodies (Table 2). The membranes were subjected to chemiluminescence using a Gel Doc EZ Imager (BIO-RAD, Hercules, CA, USA), and the images were analyzed with the Image Lab software 4.1 (BIO-RAD, Hercules, CA, USA).

### 4.5. Quantitative Succinylome Analysis

#### 4.5.1. Protein Extraction and Trypsin Digestion

*EP300* WT H1975 cells and *EP300* KO H1975 cells were subjected to the 4D-label-free-based quantitative succinylproteomic analysis (PTM Bio, Hangzhou, China). As described in the previous study [66], samples were sonicated, lysed, and centrifuged to obtain the supernatant, and its protein concentration was measured. After precipitation with trichloroacetic acid and washing with prechilled acetone, protein samples were reduced with dithiothreitol, alkylated with iodoacetamide, and digested with trypsin to obtain peptides.

#### 4.5.2. Affinity Enrichment

To enrich modified peptides, tryptic peptides dissolved in NETN buffer (100 mM NaCl, 1 mM EDTA, 50 mM Tris-HCl, 0.5% NP-40, pH 8.0) were incubated with pre-washed antibody beads (PTM Bio, PTM-402) at 4 °C overnight with gentle shaking. Then, the beads were washed, and the bound peptides were eluted from the beads. After the eluted fractions were combined and vacuum-dried, the resulting peptides were desalted with C18 ZipTips (Millipore) and separated by NanoElute ultra-high performance liquid system according to the manufacturer’s instructions.

#### 4.5.3. LC-MS/MS Analysis

LC-MS/MS analysis was performed as described in the previous study [67]. Briefly, the peptides were subjected to a capillary source, followed by the timsTOF Pro (Bruker Daltonics, Billerica, MA, USA) mass spectrometry analysis. The electrospray voltage of 1.70 kV was applied. Precursors and fragments were analyzed at the TOF detector, with an MS/MS scan range from 100 to 1700 *m*/*z*. The timsTOF Pro was operated in parallel accumulation serial fragmentation (PASEF) mode. Precursors with charge states 0 to 5 were selected for fragmentation, and 10 PASEF-MS/MS scans were acquired per cycle. The dynamic exclusion window of 30 s was applied.

#### 4.5.4. Database Search

The resulting MS/MS data were processed using the MaxQuant search engine (v.1.6.15.0, https://www.maxquant.org/, accessed on 30 June 2022). Tandem mass spectra were searched against the human SwissProt database (20,422 entries) concatenated with the reverse decoy database. Trypsin/P was selected as a cleavage enzyme, allowing for up to 2 missed cleavages. The mass tolerance for precursor ions was set as 20 ppm for the initial search and 5 ppm for the main search, while the mass tolerance for fragment ions was set to 0.02 Da. Carbamidomethyl on Cys was set as a fixed modification, and acetylation on protein N-terminal and oxidation on Met were specified as variable modifications. FDR was adjusted to <1%.

#### 4.5.5. Quantitative Analysis

Based on the signal intensity values (*I*) of each peptide in different samples, the relative quantitative values of modification sites (*R*) were calculated by the following steps: *R_ij_* = *I_ij_*/Mean(*I_j_*), *i* represents the samples, *j* represents the peptides.

#### 4.5.6. Bioinformatics Methods

(1) The subcellular localization of the identified proteins was predicted by the WoLF-PSORT database [68]; (2) The functional analysis was classified using Gene Ontology (GO, http://www.geneontology.org, accessed on 30 June 2022) Terms and UniProt-GOA database [69]; (3) The pathway mapping was employed through the Kyoto Encyclopedia of Genes and Genomes (KEGG, https://www.kegg.jp, accessed on 30 June 2022) database; (4) The domain functional descriptions were annotated by InterProScan (a sequence analysis application) [70]. Moreover, the enrichment-based clustering of the identified proteins was finished by one-way hierarchical clustering (Euclidean distance, average linkage clustering); (5) The protein–protein interaction (PPI) was analyzed using the STRING (V.11.0, https://string-db.org/, accessed in 30 June 2022) database; (6) The motif analysis was performed by MoMo analysis tool based on the motif-x algorithm [71]. The peptide sequences of 10 amino acids in each upstream and downstream of the identified modification sites were collected, and a score (DS, from MoMo tool [71]) was given for the frequency change of amino acids. DS is calculated as follows:

DS = −Log_10_^(*p*.value)∗sign(diff.percent)^

### 4.6. Targeted Metabolomics

Targeted metabolomics analysis was performed with tumor tissues from the *EP300*-WT and *EP300*-KO tumor-bearing mice models by METWARE (Wuhan, China). Briefly, 0.05 g of the sample was mixed with 500 µL of 70% methanol/water. The sample was then vortexed, centrifuged, and transferred through the Protein Precipitation Plate for further analysis. The sample extracts were analyzed using an LC-ESI-MS/MS system.

Linear ion trap (LIT) and triple quadrupole (QQQ) scans were acquired on a triple quadrupole–linear ion trap mass spectrometer (QTRAP), controlled by Analyst 1.6.3 software (Sciex, Shanghai, China). Multiquant 3.0.3 software (Sciex) was used to quantify all metabolites.

Unsupervised PCA was performed by statistics function prcomp within R. Both hierarchical cluster analysis (HCA) and Pearson Correlation Coefficients (PCC) were carried out by R package pheatmap. Significantly regulated metabolites between groups were determined by variable importance in projection (VIP) and absolute Log_2_FC (fold change). VIP values were extracted from the Orthometric Partial Least Squares-Discriminant Analysis (OPLS-DA) result and generated using the R package MetaboAnalystR. Identified metabolites were annotated using the KEGG compound database.

### 4.7. Tests of Glucose Uptake, LDH Activity, and Lactic Acid Export

Cells were cultured at 37 °C under 5% CO_2_ for 24 h. Cell lysates or cell supernatants were analyzed with the glucose assay kit (F006-1-1, Jianchengbio), the LDH assay kit (A020-1-2, Jianchengbio), and the lactic acid assay kit (A019-2-1, Jianchengbio), according to the manufacturer’s protocols.

### 4.8. Seahorse Analysis

Seahorse analyses were performed on a Seahorse XF Analyzer with the Seahorse XF RPMI Assay Medium Pack (Agilent, 103681-100) and the Seahorse XF Glycolysis Stress Test Kit (Agilent, 103020-100) according to the manufacturer’s instructions. Briefly, cells were plated in a 96-well assay plate the day before the analysis in the complete medium to ensure 80–90% confluence on the next day, then washed and incubated in a freshly prepared XF assay medium. Final glycolysis (ECAR, Extracellular Acidification Rate) was normalized to total cell numbers.

### 4.9. Proliferation, Migration, and Invasion Assays

In the CCK-8 assay, *EP300*-WT and *EP300*-KO cells (A549 or H1975) were seeded into 96-well plates suspended in CCK-8 solution (10 μL) at a 1:10 dilution ratio with serum-free RPMI-1640 (100 μL). After incubation for an additional hour, the absorbance was automatically measured at 450 nm with a microplate reader (Epoch BioTek, Agilent, Santa Clara, CA, USA).

In the colony formation assay, A549 cells (*EP300*-WT or *EP300*-KO) were seeded in a 6-well plate at 500 cells per well. After approximately 14 days, when colonies had formed, cells were fixed for 30 min and stained with 0.2% crystal violet for 10 min, rinsed with PBS buffer three times, and imaged.

The scratch test was conducted in a 6-well plate seeded by H1975 cells (*EP300*-WT or *EP300*-KO, 3 × 10^5^ cells per well). Once the cells were completely attached to the wall, scratches were made evenly and quickly. Then, the cells were gently rinsed with PBS buffer and cultured with 1% FBS-containing RPMI-1640 medium. After 24 h incubation, the cells could be observed and recorded using a microscope.

Transwell invasion assay was performed using a Transwell chamber (Millipore, PTEP24H48) coated with Matrigel gel (3432-010-01, R&D Systems, Minneapolis, MN, USA). A549 cells (*EP300*-WT or *EP300*-KO, 1 × 10^5^) in 100 μL serum-free medium were seeded in the upper chamber. The lower chamber was filled with a 10% FBS-containing RPMI 1640 medium. After 48 h of incubation, cells were washed with PBS buffer, fixed with 5% glutaraldehyde for 30 min, and stained with 0.2% crystal violet for 10 min. The invaded cells were rewashed with PBS buffer and counted.

### 4.10. LUAD Nude Mouse Models

BALB/c nude mice, male, 6–8 weeks of age, which were purchased from Beijing HFK Bioscience (Beijing, China), were selected as experimental subjects. These mice are with T cell immunodeficiency and in good health with no infection, suitable for in vivo tumorigenicity experiments. The mice were housed in a standard animal laboratory (specific pathogen-free facility) under constant environmental conditions, including a 12-h light and dark cycle and 22–25 °C housing temperature with free access to water and food.

*EP300* KO A549 cells (2.5 × 10^7^ cells/mL in 0.1 mL culture medium) were injected subcutaneously in nude mice. One week after injection, the tumors were well established, and their volumes were measured every other day. Once a tumor reached a maximum diameter of more than 15 mm, all tumors were removed from the mice. The tumor volume and weight were measured and subjected to statistical analysis.

### 4.11. IP and Co-IP

IP and co-IP were performed using the Pierce Crosslink Immunoprecipitation Kit (26147, Thermo Fisher Scientific, Waltham, MA, USA) and the Pierce Co-Immunoprecipitation Kit (26149, Thermo Fisher Scientific, Waltham, MA, USA), respectively. The protocols provided by the manufacturer were followed. Briefly, 10 μg of certain antibodies were crosslinked onto a Protein A/G resin. The antibody–agarose beads were mixed with the lysate, and the mixture was incubated with gentle mixing at 4 °C overnight. After washing with ice-cold IP lysis/wash buffer six times, the immunoprecipitated proteins were then eluted and detected via Western blotting. Anti-*ALDOA* (Proteintech, 11217-1-AP), anti-*PGK1* (Proteintech, 17811-1-AP), and anti-*PKM* (Proteintech, 25659-1-AP) antibodies were coupled to the resin for IP (Table 2). Anti-p300 (Santa Cruz, sc-48343) antibody was used for antibody immobilization for coIP (Table 2).

### 4.12. Immunohistochemistry Analysis

Immunohistochemistry was performed on serial tissue microarrays of LUAD purchased from Shanghai Outdo Biotech with a non-biotin detection system (General Two-Step Test Kit, ZSGB-BIO, PV-9000). Paraffin sections were dewaxed and hydrated, followed by an antigen retrieval step with citrate buffer (pH 6.0). After washing with PBS, the sections were treated with endogenous peroxidase blockers for 10 min and washed again with PBS buffer. Then, the sections were incubated with the anti-p300 antibody (Santa Cruz, sc-48343) at 4 °C overnight. After washing, the sections were incubated with the reaction-enhanced reagent and the HRP labeled Goat Anti-Mouse/Rabbit IgG, following the instructions of the manufacturer. Finally, 3,30-diaminobenzidine was used for the chromogenic reaction, and hematoxylin was used to counterstain the nuclei. Cells were imaged on a confocal microscope using Nikon NIS-Elements software 4.2 (Nikon, Tokyo, Japan).

The tissue microarrays were analyzed by two senior pathologists, who independently evaluated the intensity of p300 dyeing (negative, 0; weak, 1; moderate, 2; strong, 3) and the fraction (%) of p300-positive cells (<5%, 0; 5–25%, 1; 25–50%, 2; 50–75%, 3; >75%, 4). The total score was calculated by multiplying the intensity with the fraction. All tumors were categorized as high or low *EP300*-expressing based on a cut-off score of 6 [72].

## 5. Conclusions

In summary, our study identified p300 as a catalytic enzyme for Ksucc through quantitative succinylome analysis. Further targeted metabolomics and functional metabolic tests indicated that p300-mediated Ksucc facilitated glycolysis via succinylating key cytosolic glycolytic enzymes, such as *PGK1*, *PKM*, *PFKL*, and *ALDOA*, and enhanced cellular glycolysis as well as subsequent development of acidic TME. Moreover, we observed the high-frequency cytoplasmic expression of p300 in tissues from individuals and noted its association with the progression of LUAD. Our findings provide new insights into the diverse functions of Ksucc in glycolysis-related pathophysiology and highlight the potential of *EP300* as a prognostic marker for LUAD patients.

## Figures and Tables

**Table 1:** Correlation of *EP300* expression and clinicopathologic features in LUAD individuals.

Clinicopathological Variables	*EP300* Expression		χ^2^	*p*
	High (*n* = 105)	Low (*n* = 38)		
Gender				
Male	65	21	0.513	0.474
Female	40	17		
Age (year)				
<60	65	21	0.513	0.474
≥60	40	17		
*PDL1* expression				
Positive	45	10	1.495	0.221
Negative	50	19		
*EGFR* expression				
Positive	17	1	4.345	0.037 *
Negative	72	29		
*ALK* expression				
Positive	21	9	0.528	0.468
Negative	72	22		
Tumor size (cm)				
<5	76	27	0.024	0.876
≥5	29	11		
Tumor differentiation				
I–II	68	22	0.673	0.412
III–IV	36	16		
T stage				
T1–T2	72	30	3.901	0.048 *
T3–T4	33	5		
N stage				
N0–N1	67	26	0.079	0.779
N2–N3	18	8		

The clinical staging of the individuals was based on the AJCC staging (7th edition). The chi-square test was used for correlation analysis, * *p* < 0.05.

**Table 2:** The reagents or resources involved in this study.

Reagent or Resource	Source	Identifier
**Antibodies**		
Mouse monoclonal anti-p300 antibody	Santa Cruz, Santa Cruz, CA, USA	sc-48343
Mouse monoclonal anti-α-tubulin antibody	Proteintech, Rosemont, IL, USA	66031-1-lg
Mouse monoclonal anti-succinyllysine antibody	PTM Bio, Hangzhou, China	PTM-419
Rabbit polyclonal anti-*ALDOA* antibody	Proteintech, Rosemont, IL, USA	11217-1-AP
Rabbit polyclonal anti-*PGK1* antibody	Proteintech, Rosemont, IL, USA	17811-1-AP
Rabbit polyclonal anti-*PKM* antibody	Proteintech, Rosemont, IL, USA	25659-1-AP
Rabbit recombinant anti-FLAG antibody	Proteintech, Rosemont, IL,USA	80010-1-RR
Goat Anti-Rabbit IgG (H+L) secondary antibody, HRP	Invitrogen, Carlsbad, CA, USA	31460
Goat Anti-Mouse IgG (H+L) secondary antibody, HRP	Invitrogen, Carlsbad, CA, USA	31430
**Critical Commercial Assays**		
Pierce Crosslink Immunoprecipitation Kit	Thermo Fisher Scientific, Waltham, MA, USA	26147
Pierce Co-Immunoprecipitation Kit	Thermo Fisher Scientific, Waltham, MA, USA	26149
Pierce BCA protein assay kit	Thermo Fisher Scientific, Waltham, MA, USA	23225
Glucose assay kit	Jianchengbio, Nanjing, China	F006-1-1
Lactate dehydrogenase assay kit	Jianchengbio, Nanjing, China	A020-1-2
Lactic acid assay kit	Jianchengbio, Nanjing, China	A019-2-1
Seahorse XF RPMI Assay Medium Pack	Agilent, Santa Clara, CA, USA	103681-100
Seahorse XF Glycolysis Stress Test Kit	Agilent, Santa Clara, CA, USA	103020-100
General Two-Step Test Kit	ZSGB Bio, Beijing, China	PV-9000
**Plasmids**		
*EP300* CRISPR/Cas9 KO plasmid	Sangon Biotech, Shanghai, China	This paper
pLV3-CMV-*PGK1*-3×FLAG-CopGFP	MiaoLing bio, Wuhan, China	This paper
pLV3-CMV-*PKM*-3×FLAG-CopGFP	MiaoLing bio, Wuhan, China	This paper
pLV3-CMV-*ALDOA*-3×FLAG-CopGFP	MiaoLing bio, Wuhan, China	This paper
pLV3-CMV-*PGK1*-K323R-3×FLAG-CopGFP	MiaoLing bio, Wuhan, China	This paper
pLV3-CMV-*PKM*-K166R-3×FLAG-CopGFP	MiaoLing bio, Wuhan, China	This paper
pLV3-CMV-*ALDOA*-K13R-3×FLAG-CopGFP	MiaoLing bio, Wuhan, China	This paper
**Biological Samples**		
Human LUAD tissue microarray	Outdo Biotech, Shanghai, China	HLugA180Su04, HLugA180Su08
**Cell Lines**		
A549	ATCC, Gaithersburg, MD, USA	CCL-185
H1975	ATCC, Gaithersburg, MD, USA	CRL-5908
**Organisms**		
BALB/c nude mice	HFK Bioscience, Beijing, China	BALB/c nude mice
**Reagents**		
Lipofectamine 2000	Invitrogen, Carlsbad, CA, USA	11668019
Blasticidin	Invitrogen, Carlsbad, CA, USA	Ant-bl-1
Ampicillin sodium	Solarbio, Beijing, China	A8180
CCK-8	Engreen, Beijing, China	EC008
Hematoxylin stain solution, Gill I	Solarbio, Beijing, China	G4492
Matrigel gel	R&D Systems, Minneapolis, MN, USA	3432-010-01
**Software and Algorithms**		
Prism 8.0	GraphPad, San Diego, CA, USA	N/A
SPSS 25.0	IBMCorp, New York, NY, USA	N/A
ImageJ 1.51	National Institutes of Health, Bethesda, MD, USA	N/A
Image Lab 6.1	BIO-RAD, Hercules, CA, USA	N/A

## Data Availability

The data in the succinylome analysis and the metabolomic analysis are available in the supplement information. The datasets used and/or analyzed during the current study are available from the corresponding author upon reasonable request.

## Supplementary Materials

The following supporting information can be downloaded at: https://www.mdpi.com/article/10.3390/ijms25021057/s1.

## Abbreviations

*ALDOA*: Fructose-Bisphosphate Aldolase A; *ALK*: Anaplastic Lymphoma Receptor Tyrosine Kinase; ECAR: extracellular acidification; GO: Gene Ontology; HCA: hierarchical cluster analysis; IP: Immunoprecipitation; Ksucc: lysine succinylation; Kac: protein lysine acetylation; KEGG: Kyoto Encyclopedia of Genes and Genomes; KOGs: Eukaryotic Orthologous Genes; LUAD: lung adenocarcinoma; Na-succ: sodium succinate; NSCLC: non-small cell lung cancer; OPLS-DA: Orthometric Partial Least Squares-Discriminant Analysis; PASEF: parallel accumulation serial fragmentation; PCA: Principal Component Analysis; *PCAF*: P300/CBP-associated factor; PCC: Pearson’s Correlation Coefficient; *PCK2*: Phosphoenolpyruvate Carboxykinase 2, mitochondrial; *PDL1*: Programmed Cell Death 1 Ligand 1; *PFKL*: Phosphofructokinase 1, liver type; *PFKM*: Phosphofructokinase 1, muscle type; *PFKP*: Phosphofructokinase 1, platelet type; *PGK1*: Phosphoglycerate Kinase 1; *PKM*: Pyruvate Kinase M1/2; PMSF: phenylmethyl-sulfonyl fluoride; PPI: protein–protein interaction; PTMs: post-translational modifications; RIPA: radio immunoprecipitation assay lysis buffer; RSD: Relative Standard Deviation; *SIRT7*: Sirtuin 7; TCA cycle: citrate cycle; TME: tumor microenvironment; VIP: variable importance in projection.
