# Supplementary material for: Acetyltransferase P300 Regulates Glucose Metabolic Reprogramming through Catalyzing Succinylation in Lung Cancer
Source: Int J Mol Sci. 2024 Jan 15;25(2):1057. doi: 10.3390/ijms25021057 (PMC10816063; doi:10.3390/ijms25021057)
Supplement: Supplementary file 1 [file ijms-25-01057-s001.zip › ijms-2694052-supplementary material.pdf]

Supplementary Information

Acetyltransferase P300 Regulates Glucose Metabolic Reprogramming through Catalyzing Succinylation in Lung Cancer

Qingzhi Ma †, Qingmei Zeng †, Kun Wang †, Meirui Qian, Jingzhuo Li, Hao Wang, Huijie Zhang, Jianli Jiang *, Zhinan Chen *, Wan Huang *

Supplementary materials include 3 figures, 1 table and 2 data files:

Figure S1. The construction and validation of *EP300* knockout cells (A549 and H1975).

Figure S2. All the samples for the succinylome analysis were validated for sample quality and repeatability.

Figure S3. The landscape of metabolites altered by p300-mediated Ksucc in the metabolomics analysis.

Table S1. The enrichment of amino acid residues in the flanking region close to p300-related Ksucc sites.

Additional file 1 Differentially regulated sites in the succinylome analysis.xlsx (provided beyond this manuscript).

Additional file 2 Metabolites identified in the metabolomic analysis.xlsx (provided beyond this manuscript).

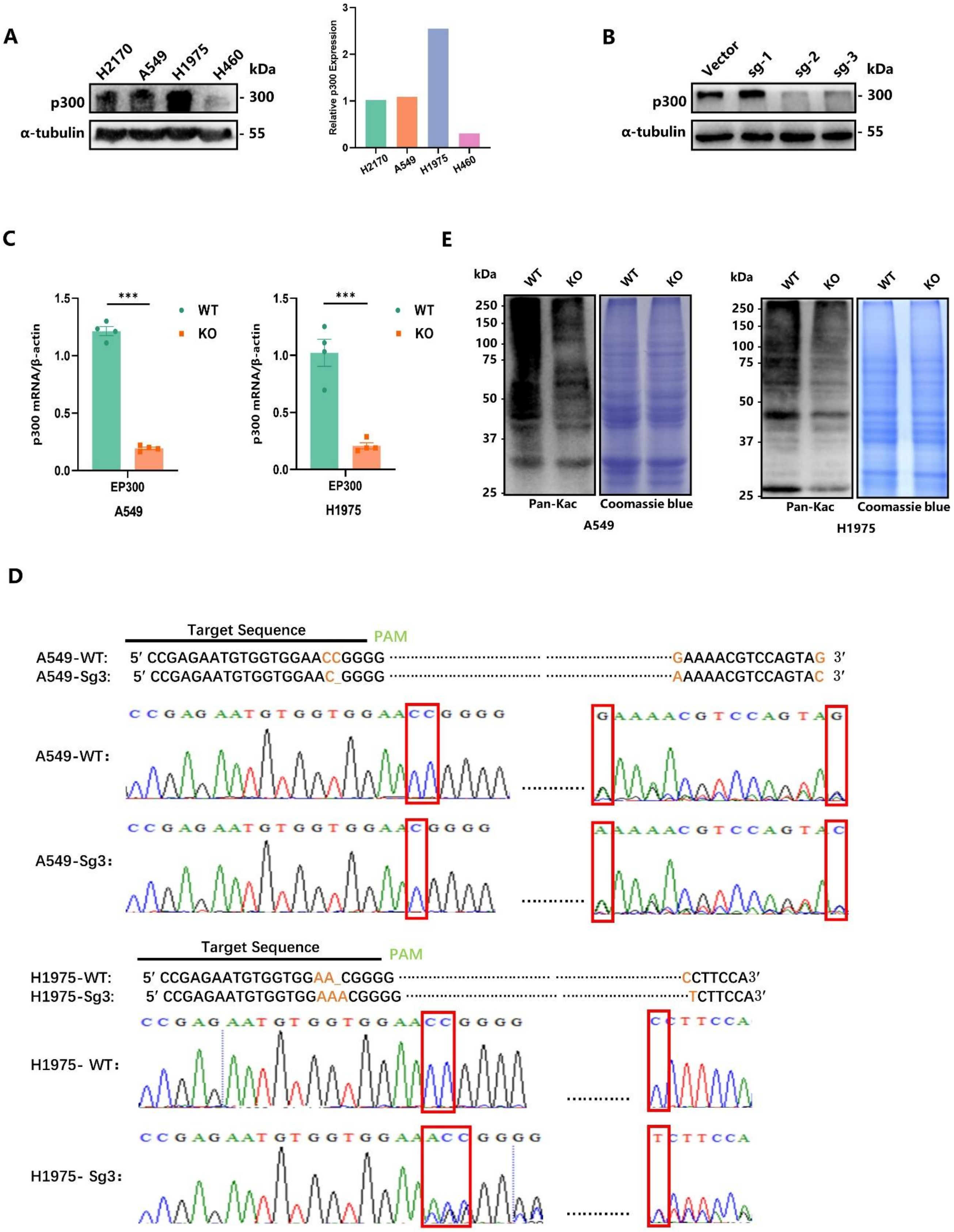

Figure S1. The construction and validation of *EP300* knockout cells (A549 and H1975). (A-B) (A) The *EP300* expression levels in the four cell lines were detected by immunoblotting with anti-p300 antibodies. The bar charts represented the gray level in the immunoblotting results and the highest levels of *EP300* expression were found in the A549 and H1975. (B) Among all *EP300*-knockout plasmids constructed, sgRNA-3 was the best. (C) The deletion of

EP300 in A549 and H1975 cells was confirmed by real-time PCR using primers as follows: forward (TCTCCAGCCACTGCGACC) and reverse (CCGATATGACATAGTGTTG). (D) Monoclonal cells of A549-*EP300*-Sg3 and H1975-*EP300*-Sg3 were selected for DNA sequencing to compare against the respective no-load control group (WT). (E) *EP300* deficiency impairs Kac levels in both A549 and H1975 cells. Kac levels in WT and *EP300* KO cells were determined by western blot with a pan-antibody.

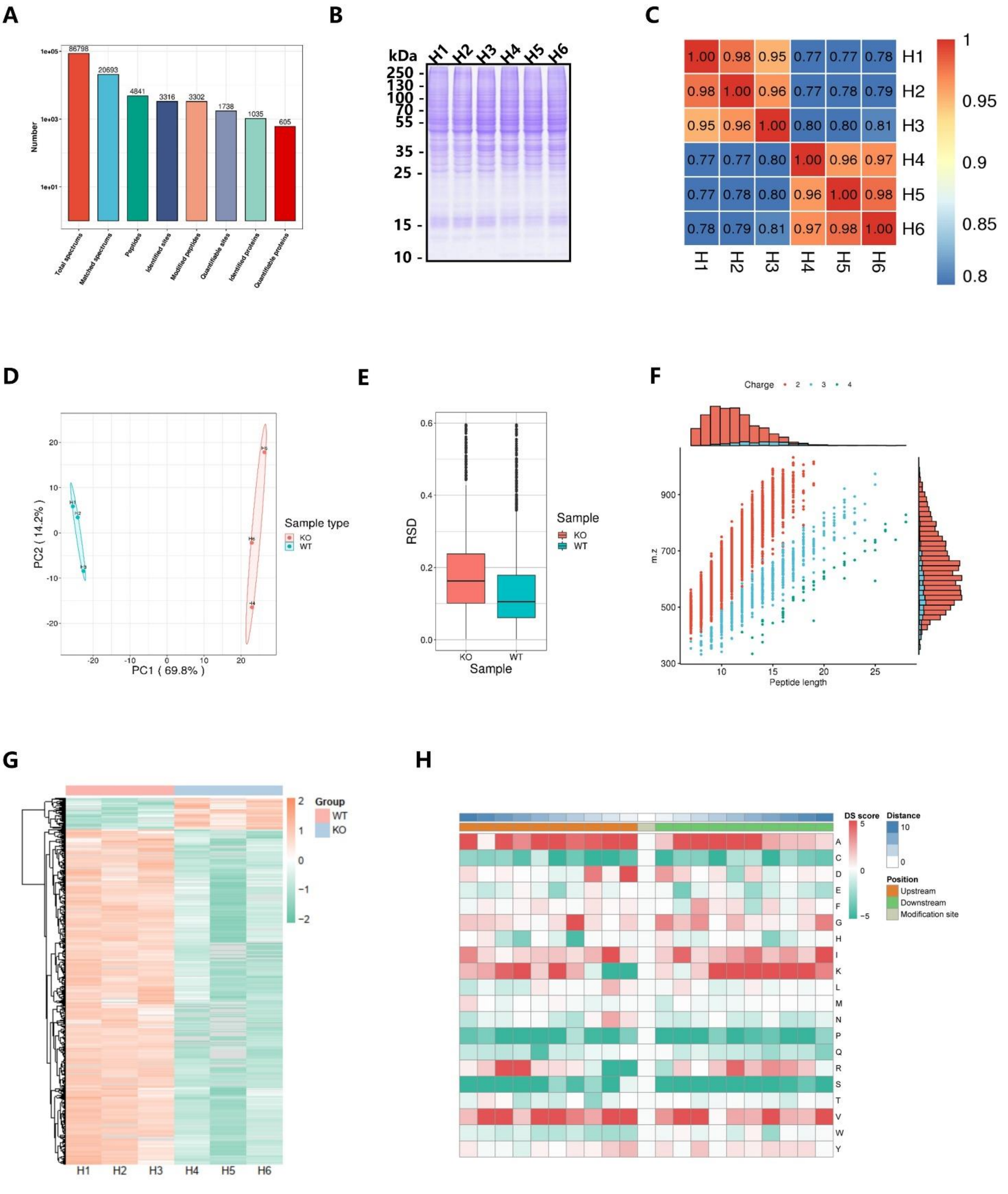

Figure S2. All the samples for the succinylome analysis were validated for quality and repeatability. (A) The bar plot shows the results of the database search. (B) The samples for LC-MS/MS analysis were confirmed by coomassie staining before application. (C-F) The samples for LC-MS/MS analysis were

also validated by assessment procedures for repeatability (including PCC, PCA, and RSD). (G) The heatmap, as a supplement to the volcano map in Figure 2C, shows the results of difference analysis and clustering between the *EP300*-WT and *EP300*-KO groups. (H) The frequency of amino acids occurring near the Ksucc sites has changed to DS scores, as shown by the heatmap.

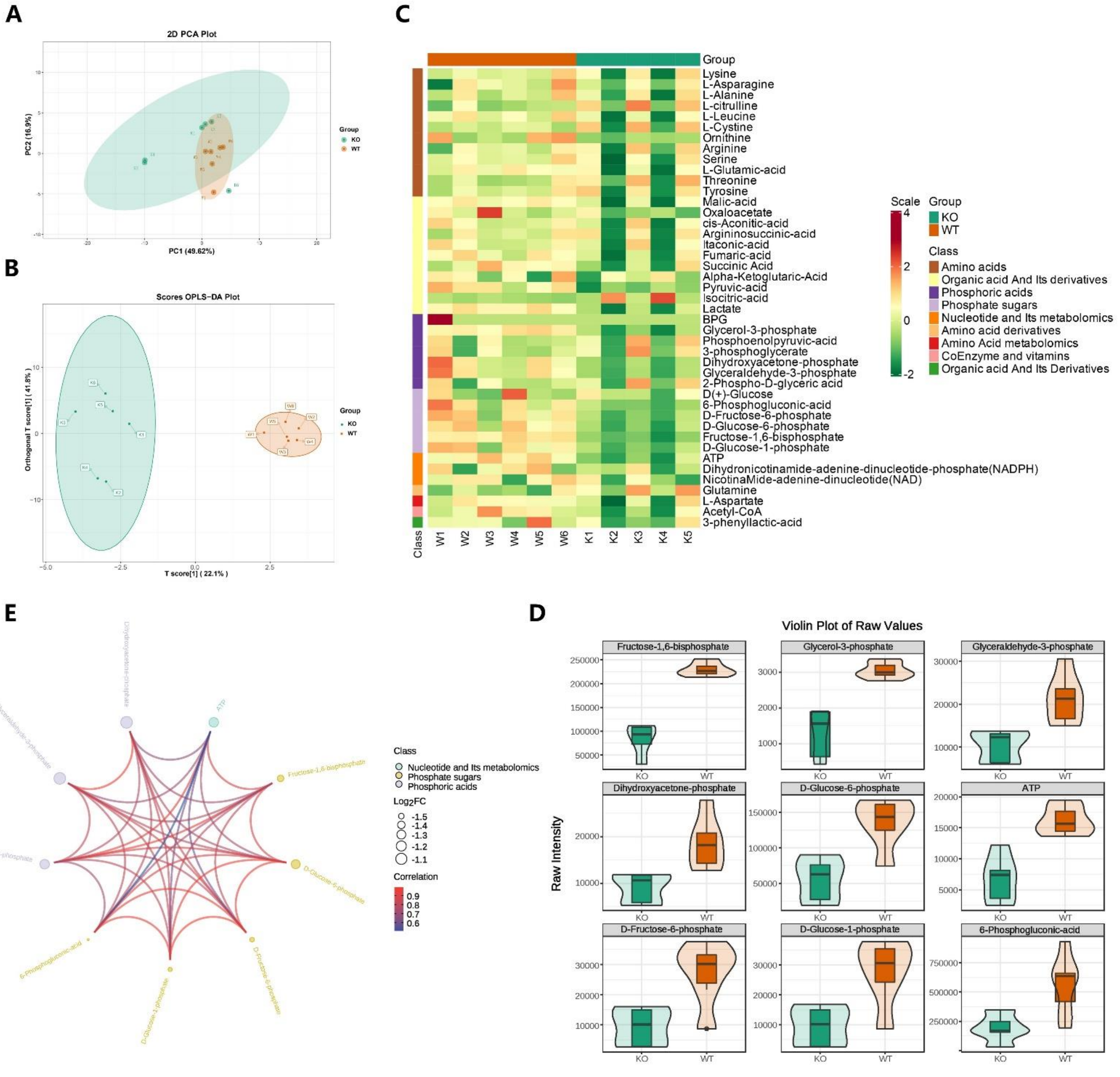

Figure S3. The landscape of metabolites altered by p300-mediated Ksucc in the metabolomics analysis. (A-B) Principal Component Analysis and Orthometric Partial Least Squares-Discriminant Analysis of two groups. PC1 represents the first principal component, PC2 represents the second principal component, and percentage represents the interpretation rate of this principal component to the data set. (C) Clustering heatmap for all metabolites. Horizontal is the sample name, vertical is the metabolite information, and different colors are the colors filled with different values obtained after standardized treatment with different relative contents (red represents high content, green represents low content). (D) Violin diagram of differential metabolites. (E) The correlation heatmap of differential metabolites. Different colors represent the level of the Pearson correlation coefficient, and the relationship between the correlation coefficient and color is illustrated in the legend on the right. Red indicates a strong positive correlation, green indicates a strong negative correlation, and the darker the color the greater the absolute value of the correlation coefficient between samples.

Table S1. The enrichment of amino acid residues in the flanking region close to p300-related Ksucc sites.

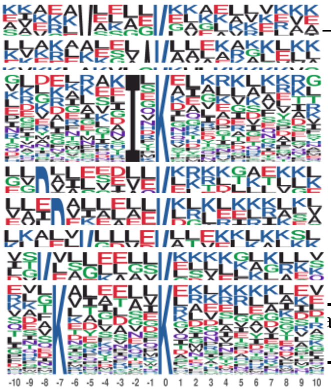

Motif Logo	Motif	Motif Score	Foreground		Background		Fold Increase
			Matches	Size	Matches	Size	
	**xxxxxVxxxx_K_xxxxxxxxxx**	**11.00**	292	3316	35474	604474	1.5
	xxxxxxxxxA_K_xxxxxxxxxx	11.81	272	3024	32974	569000	1.6
	xxxxxxxxxx_K_xVxxxxxxxx	9.09	236	2752	30601	536026	1.5
	Axxxxxxxxx_K_xxxxxxxxxx	7.92	234	2516	32313	505425	1.5
	xxxxxxxxIx_K_xxxxxxxxxx	8.83	178	2282	23125	473112	1.6
	xxRxxxxxxx_K_xxxxxxxxxx	8.66	181	2104	24532	449987	1.6
	xxxRxxxxxx_K_xxxxxxxxxx	7.53	162	1923	22964	425455	1.6
	xxxxxKxxxx_K_xxxxxxxxxx	6.90	213	1761	34044	402491	1.4
	xxKxxxxxxx_K_xxxxxxxxxx	7.77	180	1548	28079	368447	1.5
	xxxKxxxxxx_K_xxxxxxxxxx	8.99	169	1368	26101	340368	1.6

[illegible] amino acids from the upstream and downstream of the modification sites were included as analytic targets. The analyses were based on the MoMo analysis tool with the Motif-X algorithm.
